# Gas embolism caused by gas-forming pyogenic liver abscess

**DOI:** 10.2478/jtim-2023-0093

**Published:** 2023-07-05

**Authors:** Weisheng Chen, Miaoxian Fang, Chunbo Chen

**Affiliations:** Department of Emergency Intensive Care Unit, Huizhou Third People’s Hospital, Guangzhou Medical University, Huizhou 516000, Guangdong Province, China; Department of Intensive Care Unit of Cardiac Surgery, Guangdong Provincial People's Hospital, Guangdong Academy of Medical Sciences, Guangdong Cardiovascular Institute, Guangzhou 510080, Guangdong Province, China; Department of Critical Care Medicine, Guangdong Provincial People's Hospital, Guangdong Academy of Medical Sciences, Guangzhou 510080, Guangdong Province, China; Department of Critical Care Medicine, Maoming People's Hospital, Maoming 525000, Guangdong Province, China; The Second School of Clinical Medicine, Southern Medical University, Guangzhou 510080, Guangdong Province, China

## To The Editor

Gas-forming pyogenic liver abscess (GFPLA), accounting for 1%–5% of pyogenic liver abscess,^[1]^ is usually associated with diabetes and abnormality of blood glucose level. *Klebsiella pneumoniae*, usually detected from blood culture and liver puncture, is the most frequent bacterium for GFPLA, with a tendency to metastatic infection and shock. Between 27.7% and 37.1% of patients could die due to this.^[2–3]^ Therefore, immediate and effective treatment is of vital importance. In case of metastatic intracranial infection caused by GFPLA, meningitis and brain abscess are the most frequent, while metastatic gas embolization and pneuomocrania are rare. Here, we present a rare case in which the patient died from cerebral gas embolization due to *K. pneumoniae*-induced GFPLA.

**Figure 1:**
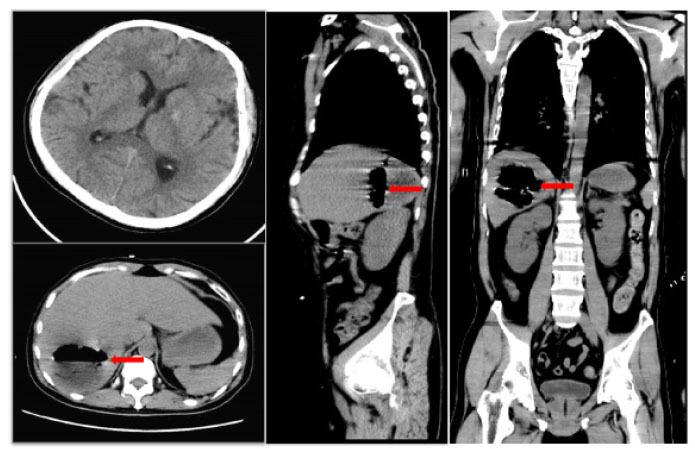
Initial brain CT scan shows no abnormalities, while sagittal and axial abdominal CT scans reveal a hepatic abscess with high gas and fluid levels in the right lobe. Red arrow: a large amount of gas is seen in the cavity of the liver abscess. CT: computed tomography.

**Figure 2:**
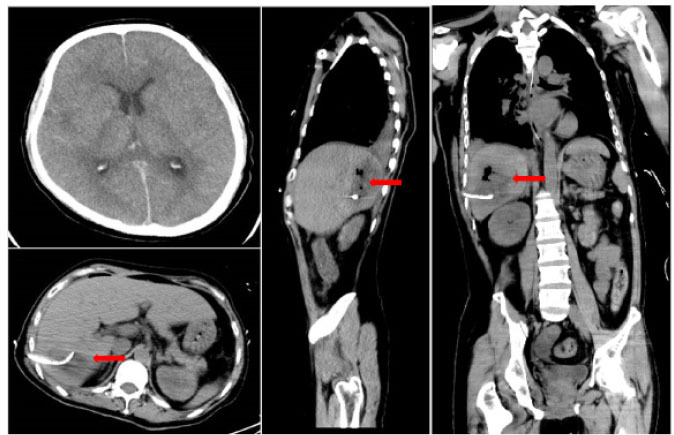
Repeat CT scans on day 2 reveal emerging cerebral edema and subarachnoid hemorrhage, but there is a decrease in hepatic gas and fluid levels. Red arrowhead: after liver abscess puncture drainage, the gas in the abscess cavity had decreased obviously. CT: computed tomography.

**Figure 3:**
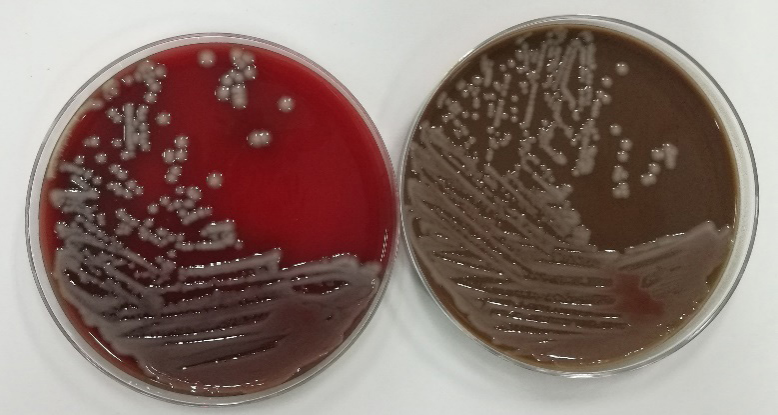
Culture of blood and abscess fluid demonstrating *Klebsiella pneumoniae* infection

**Figure 4:**
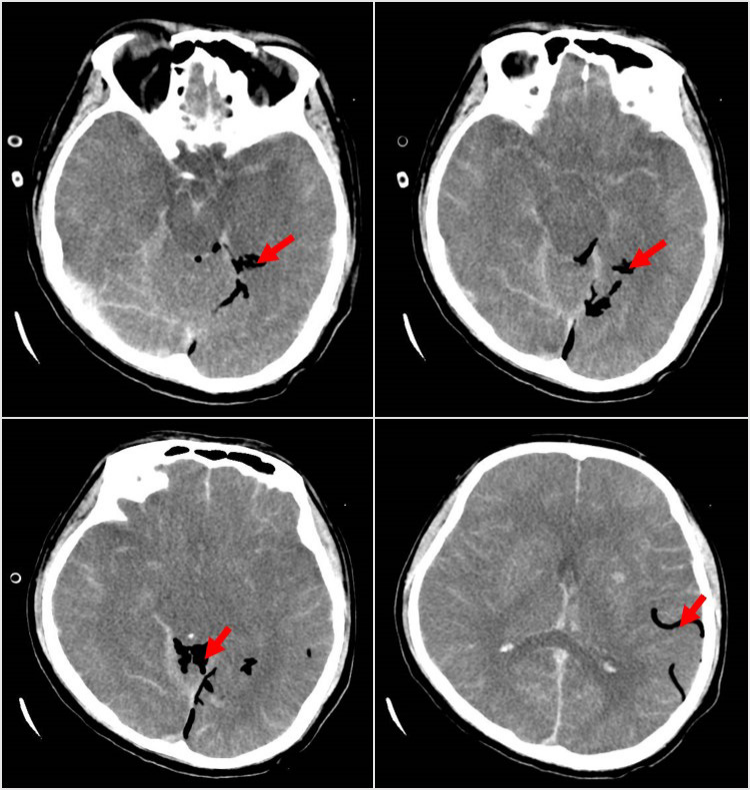
CT scan on day 5 showing exacerbated brain swelling, cerebral herniation, and emerging pneumocrania. Red arrow: visible intracranial pneumatosis. CT: computed tomography.

A 41-year-old male was admitted to the intensive care unit with sustained chills and fevers for 1 week and coma for 5 h. Leukocyte, neutrophil, C-reactive protein, and procalcitonin levels were high and platelet level was low. There was a significant increase in glycosylated hemoglobin, indicating undiagnosed diabetes mellitus or poor glucose control. Computed tomography (CT) revealed liver abscess with a large fluid–gas plane, but indicated no brain abnormalities (Figure 1). A diagnosis of GFPLA was made; percutaneous abscess drainage combined with anti-infective therapy was carried out, but the patient’s condition deteriorated, and bilateral pupil dilatation occurred on the next day. Immediate CT review showed brain edema and subarachnoid hemorrhage despite decrease in hepatic fluid and gas levels (Figure 2). A culture of abscess fluid and blood, along with next-generation sequencing yielded a definitive diagnosis of *K. pneumoniae* infection (Figures 3). Anaerobic and fungal cultures were negative. Anti-infective therapy based on drug-sensitive experiments was administered (imipenem cilastatin 2 g, every 6 hours), but the treatment was not effective and the patient eventually died of progressive cerebral swelling, herniation, and pneuomocrania (Figure 4).

GFPLA is the extreme condition of pyogenic liver abscess accounting for 1%– 5% of all liver abscesses, and is associated with higher morbidity and mortality than non–gas-forming pyogenic liver abscess.^[1]^ The majority of GFPLA cases are infected by *K. pneumoniae* with a high incidence of extrahepatic complications involving eye, muscle, fascia, and the central nervous system.^[4–5]^ Patients with extrahepatic metastatic infection have a relatively worse condition and are more often admitted to intensive care unit (ICU). Nevertheless, almost 20% of patients will die eventually.^[2]^

In this case, the patient developed a metastatic intracranial infection and pneumocephalus, which led to severe brain edema and hernia. This is rare, but a life-threatening complication and is worth being concerned about.

GFPLA is mainly diagnosed via ultrasound (US) or a computerized tomography (CT) scan. Lee *et al*.^[3]^ reported 100% detection rate through either US or CT. Relevant studies have shown that monomicrobial *K. pneumoniae* was the most common bacterium found in patients with GFPLA.^[3,6]^ *K. pneumoniae* was found in 85.9% (80.6%–100%) of positive liver pus cultures in GFPLA patients versus 67.7% (65.8%–85.7%) in non-GFPLA patients. In this case, the pus cavity and blood culture of the patient were confirmed to be *K. pneumoniae*, but the cerebrospinal fluid culture could not be performed due to the existence of cerebral hernia. Therefore, the intracranial metastasis of *K. pneumoniae* could not be directly confirmed. However, recent studies have indicated that *K. pneumoniae*-induced GFPLA is significantly associated with metabolic disorders including hypertension, diabetes, and fatty liver. Poorly controlled diabetes is probably an independent risk factor for extrahepatic complications.^[7]^ Glucose fermentation by bacteria causes the accumulation of formic acid, which is metabolized by formic hydrogenlyase. Gas is produced and accumulated in the process, and large amount of gas accumulation will inevitably lead to the continuous increase of pressure in the abscess cavity, which will subsequently cause difficulty of drug entry and the high risk of bacterial blood entry.^[3]^ In spite of the absence of history of diabetes in this case, a significantly increased glycosylated hemoglobin and glycosylated albumin suggested undiagnosed diabetes, and poor glucose control may be the main risk factor for dissemination. Therefore, the patient in this case had a high risk of distant spread of infection, and new intracranial pneumatosis could be seen on the head CT, reexamined on the fifth day after admission. It is not ruled out that pathogenic bacteria and gas enter the blood due to high pressure in the pus cavity. While considering the impact of intracranial infection caused by *K. pneumoniae*, we should also pay attention to the impact of air embolism on the blood supply of brain tissue, which is bound to cause ischemia, hypoxia, and the swelling of brain tissue. In terms of treatment, in addition to antibiotic and abscess drainage, for the GFPLA patients, attention must be paid on strict glycemic monitoring and control in order to prevent metastatic infection. Furthermore, GFPLA patients could have impaired consciousness due to toxic infectious materials, which must be differentiated from metastatic intracranial infection through immediate image scanning to avoid a delay in treatment.
